# Cardiovascular Outcomes in Sleep-Disordered Breathing: Are We Under-estimating?

**DOI:** 10.3389/fneur.2022.801167

**Published:** 2022-03-15

**Authors:** Muhammad Yasir, Amina Pervaiz, Abdulghani Sankari

**Affiliations:** ^1^Ascension Providence Hospital, Southfield, MI, United States; ^2^Department of Internal Medicine, Michigan State University, East Lansing, MI, United States; ^3^Department of Internal Medicine, Wayne State University, Detroit, MI, United States; ^4^Director of Medical Education/Designated Institutional Official (DIO), Ascension Providence Hospital, Southfield, MI, United States

**Keywords:** obstructive sleep apnea, sleep-disordered breathing, precision medicine, nocturnal heart rate changes, cardiovascular disease, heart failure, coronary artery disease, positive airway pressure

## Abstract

Obstructive sleep apnea is a growing health concern, affecting nearly one billion people worldwide; increasingly recognized as an independent cardiovascular risk factor associated with incident obesity, insulin resistance, hypertension, arrhythmias, stroke, coronary artery disease, and heart failure. The prevalence of obstructive sleep apnea could be underestimated in the previous studies, leading to only modest predictions of cardiovascular outcomes. Using more physiologic data will increase sensitivity for the diagnosis of obstructive sleep apnea. Individuals at high risk of obstructive sleep apnea should be identified so that treatment efforts can be focused on them. This review will assess the evidence for the relationship between obstructive sleep apnea and cardiovascular consequences in the past, present, and future. We will also explore the role of adding physiological data obtained from sleep studies and its ability to enhance the cardiovascular outcome's predictability. Finally, we will discuss future directions and gaps that need further research.

## Introduction

Obstructive sleep apnea (OSA) is one of the most common chronic diseases, affecting nearly one billion people worldwide and significantly burdens individuals and society (1). OSA affects at least 2–6% of the U.S. population (2) with a higher prevalence in men than women by 2:1 in population-based studies and up to 8:1 in referral populations (3, 4). Furthermore, when more stringent definitions are used (e.g., AHI ≥5 events per hour plus symptoms or AHI ≥15 events per hour), the estimated prevalence is ~15 percent in males and 5 percent in females (5, 6). However, the risk is similar once women are peri and post-menopausal (7).

OSA is characterized by obstructive apneas, hypopneas, and/or respiratory effort-related arousals caused by repetitive collapse of the upper airway during sleep. This presence is most reliably shown by attended overnight polysomnography in a sleep laboratory. Sleep stages, arterial oxyhemoglobin saturation, respiratory movements of the rib cage and abdomen, respiratory effort, or both, are recorded (8). Apnea-hypopnea index (AHI) is calculated by adding all of the apneas and hypopneas during the scored sleep time and dividing by total sleep time in hours. OSA is defined as an AHI of more than five events per hour of sleep (2, 6).

## Cardiovascular Consequences of OSA the Past, the Present and the Future: The Past

Several extensive studies linked OSA to increased cardiovascular morbidity and mortality (6, 9–12). Untreated moderate to severe obstructive sleep apnea is linked to hypertension, cardiac arrhythmias, coronary artery disease, and congestive heart failure. Although OSA is two to four times more common in men, relationships of biomarkers of myocardial injury and incident heart failure related to OSA appear to be stronger in women than in men (13).

Positive airway pressure (PAP) is regarded as the cornerstone of treatment for OSA (14–17). However, there is uncertainty regarding the benefits of treatment of OSA in reducing the risk of cardiovascular events such as myocardial infarction, hospitalization from heart failure, unstable angina, or cardiovascular deaths. Several observational studies and clinical trials in the past decade have been designed to study the effect of PAP therapy on cardiovascular outcomes.

In an observational study, Marin et al. showed that men with severe OSA benefited from PAP treatment and had a reduction in the number of fatal (death from myocardial infarction or stroke) and non-fatal cardiovascular events (occurrence of non-fatal myocardial infarction, stroke, and acute coronary insufficiency that needed coronary artery bypass surgery or percutaneous transluminal coronary angiography) (18).

In a randomized controlled study with a similar patient population of non-sleepy patients with OSA and without established cardiovascular disease, the CPAP therapy didn't result in a statistically significant reduction in the incidence of hypertension or cardiovascular events in patients treated with CPAP therapy (19). Similar results were noted by Peker et al. in another randomized controlled trial with no significant reduction in the long-term adverse cardiovascular outcomes in patients with known coronary artery disease (CAD) and non-sleepy patients with OSA who were treated with CPAP therapy (20).

The Sleep Apnea Cardiovascular Endpoints (SAVE) is one of the largest multicenter randomized clinical trials that studied the effect of PAP therapy on cardiovascular outcomes (21). In this secondary prevention trial, patients with moderate to severe OSA and established cardiovascular disease were randomized to CPAP therapy plus usual care or usual care alone and followed for 3.7 years. There was a significant reduction in the AHI from 29 to 3.7 events per hour per night, showing adequate control of OSA. However, CPAP therapy was not associated with a significant reduction in cardiovascular events. Although the rate of cardiovascular events was slightly improved in those adherents to CPAP (i.e., ≥4 h per night), the benefit was not statistically significant. Limitations of the study include the exclusion of high-risk patients: patients with “sleepy” OSA like the previous randomized trials, patients at high risk of an accident, and severe hypoxemia. In addition, there was a short follow-up period (3.7 years only) and overall low adherence to CPAP with a mean of 3.3 h per night, which may have also reduced the impact of the therapy on the cardiovascular outcome. It can be postulated from these trials that failure of PAP therapy to demonstrate the protective effects on the cardiovascular outcomes and vascular deaths could be due to the limited adherence to the treatment and short follow-up period in many trials. Furthermore, it is unclear if the “sleepy OSA” group of patients will benefit from treatment with PAP therapy as most of the trials included non-sleepy OSA patients, and longer follow-up period 5–10 years (19, 20).

The meta-analysis of 10 randomized trials by Yu et al. revealed that there was no significant association between PAP treatment and a range of cardiovascular events such as acute coronary events, stroke, or vascular death. Although, based on the available evidence, it is reasonable to recommend PAP therapy to improve symptoms in patients with OSA but not for the protection against vascular disease or death. The data also emphasizes the importance of proven treatments, such as blood pressure-lowering, lipid-lowering, and antiplatelet therapy in patients with sleep apnea, who should be treated according to established guidelines for patients at elevated cardiovascular risk (22).

While cross-sectional studies report a significant relationship between OSA and cardiac consequences, prospective (both community and clinic-based) studies provide mixed results (as depicted in Table 1). Several factors may influence the association of OSA with incident coronary heart disease in patients with OSA, such as (1) poor adherence to CPAP treatment (23) and associated comorbidities (24), (2) the severity of and presence of significant sleepiness, which are most likely to benefit from CPAP were either under-represented (severe disease) or excluded (severe sleepiness) (25), (3) relatively short follow up for the mortality end point (mean follow up 3.7 years in the SAVE trial). For example, in Punjabi et al. study on sleep disordered breathing mortality the average follow up of 8 years allowed to find a significant effect of OSA on all-cause mortality in only in men 40–70 years old who had severe disease (9). Likewise, more recent analysis of the Wisconsin cohort using heart rate data measurements and follow up for up to 15 years allowed to find significant effect of frequent heart rate changes on cardiac related mortality and morbidity, in men but not in women, and remained significant after adjusting for demographic factors, AHI 4%, hypoxemia and other comorbidities (26). Therefore, the effect of PAP therapy on different subgroups of patients with OSA needs to be better defined.

**Table 1:** Association of positive airway pressure with cardiovascular events and deaths in the randomized clinical trials.

	**Positive airway pressure**		**Control**		**Risk ratio**
	**Events or deaths, No**.	**Participants, No**.	**Events or deaths, No**.	**Participants, No**.	**(95% CI)**
**Major adverse cardiovascular events**					
Barbé et al. (19)	6	357	10	366	0.62 (0.23–1.67)
Peker et al. (20)	17	122	21	122	0.81 (0.45–1.46)
McEvoy et al. (21)	134	1,359	127	1,358	1.05 (0.84–1.33)
**Major adverse cardiovascular events plus**					
Barbé et al. (19)	23	357	21	366	1.12 (0.63–1.99)
Peker et al. (20)	47	122	53	122	0.89 (0.66–1.20)
McEvoy et al. (21)	233	1,359	217	1,358	1.07 (0.91–1.27)
**Cardiovascular deaths**					
Barbé et al. (19)	1	357	0	366	3.08 (0.13–75.24)
Peker et al. (20)	3	122	7	122	0.43 (0.11–1.62)
McEvoy et al. (21)	25	1,359	20	1,358	1.25 (0.70–2.24)
**All-cause death**					
Barbé et al. (19)	8	357	3	366	2.73 (0.73–10.22)
Peker et al. (20)	7	122	9	122	0.78 (0.30–2.02)
McEvoy et al. (21)	40	1,359	43	1,358	0.93 (0.61–1.42)
**Non-Cardiovascular deaths**					
Barbé et al. (19)	7	357	13	366	0.55 (0.22–1.37)
Peker et al. (20)	4	122	2	122	2.00 (0.37–10.72)
McEvoy et al. (21)	15	1,359	23	1,358	0.65 (0.34–1.24)
**Acute coronary syndromes**					
Barbé et al. (19)	2	357	8	366	0.26 (0.05–1.20)
Peker et al. (20)	11	122	8	122	1.38 (0.57–3.30)
McEvoy et al. (21)	42	1,359	39	1,358	1.08 (0.70–1.65)
**Stroke**					
Barbé et al. (19)	3	357	2	366	1.54 (0.26–9.15)
Peker et al. (20)	3	122	6	122	0.50 (0.13–1.95)
McEvoy et al. (21)	67	1,359	68	1,358	0.98 (0.71–1.37)
**Hospitalization for unstable angina**					
Barbé et al. (19)	17	357	11	366	1.58 (0.75–3.34)
McEvoy et al. (21)	99	1,359	5	659	1.10 (0.83–1.45)
**Heart failure**					
Barbé et al. (19)	3	357	5	366	0.62 (0.15–2.55)
Peker et al. (20)	30	122	32	122	0.94 (0.61–1.44)
McEvoy et al. (21)	17	1,359	17	1,358	1.00 (0.51–1.95)

Major adverse cardiovascular events: consist of cardiovascular death, non-fatal acute coronary syndrome, and non-fatal stroke. Major adverse cardiovascular events plus: indicates major adverse cardiovascular events in addition to hospitalization for unstable angina.

## The Present

Several recent large studies have looked to the effect of PAP therapy on cardiovascular outcome in patients with OSA with conflicting results. The SAVE and ISAACC studies contribute to understanding the observed effect of CPAP treatment for secondary cardiovascular prevention. In a randomized control trial, CPAP in patients with Acute Coronary Syndrome (ACS) and OSA (ISAACC) by Sánchez-de-la-Torre et al. patients with ACS and OSA, CPAP treatment did not result in a significantly lower incidence of cardiovascular events (27). This study excluded patients with daytime sleepiness, mean follow-up for 3.35 years, and adherence 2.78 h/night, which are significant limitations for this study.

The effect of OSA on cardiovascular events in different ACS subgroups was investigated by Zapater et al. (28) in a *post-hoc* analysis of the ISAACC study. The study indicates a significant effect of moderate-severe OSA on the risk of recurrent CV events observed only in patients in the “no-previous CVD” subgroup with an adjusted hazard ratio (HR) of 1.54 [95% confidence interval (CI), 1.06–2.24]. On the contrary, this effect was not observed in patients in the “previous CVD” subgroup (adjusted HR, 0.69; 95% CI, 0.46–1.04); Suggesting that OSA is associated with an increased risk of recurrent cardiovascular events.

In an analysis from a long-term observational cohort study, a PAP prescription was associated with 42% lower mortality among patients with severe OSA, but this risk reduction was not seen until 6–7 years of follow-up (29). The adjusted HR of all-cause mortality for participants prescribed PAP relative to those who were not was 0.38 (95% CI, 0.18–0.81). The secondary analysis further suggests that prescription of PAP therapy might also be associated with a lower risk of CV mortality. Thus, randomized controlled trials with longer follow-up and focus on high-risk patients with severe symptomatic OSA are needed to clarify the clinical benefits of PAP therapy.

### Variables Mediate the Relationship Between OSA and CVD (Figure 1)

#### Excessive Sleepy Subtype

Recently, Mazzotti et al. (30) showed that excessively sleepy subtype was associated with more than a 3-fold increased risk of heart failure. However, the excessive sleepiness phenotype may be a marker of underlying cardiovascular risk pathways influenced by OSA severity but not an independent risk factor in the absence of elevated AHI.

**Figure 1:**
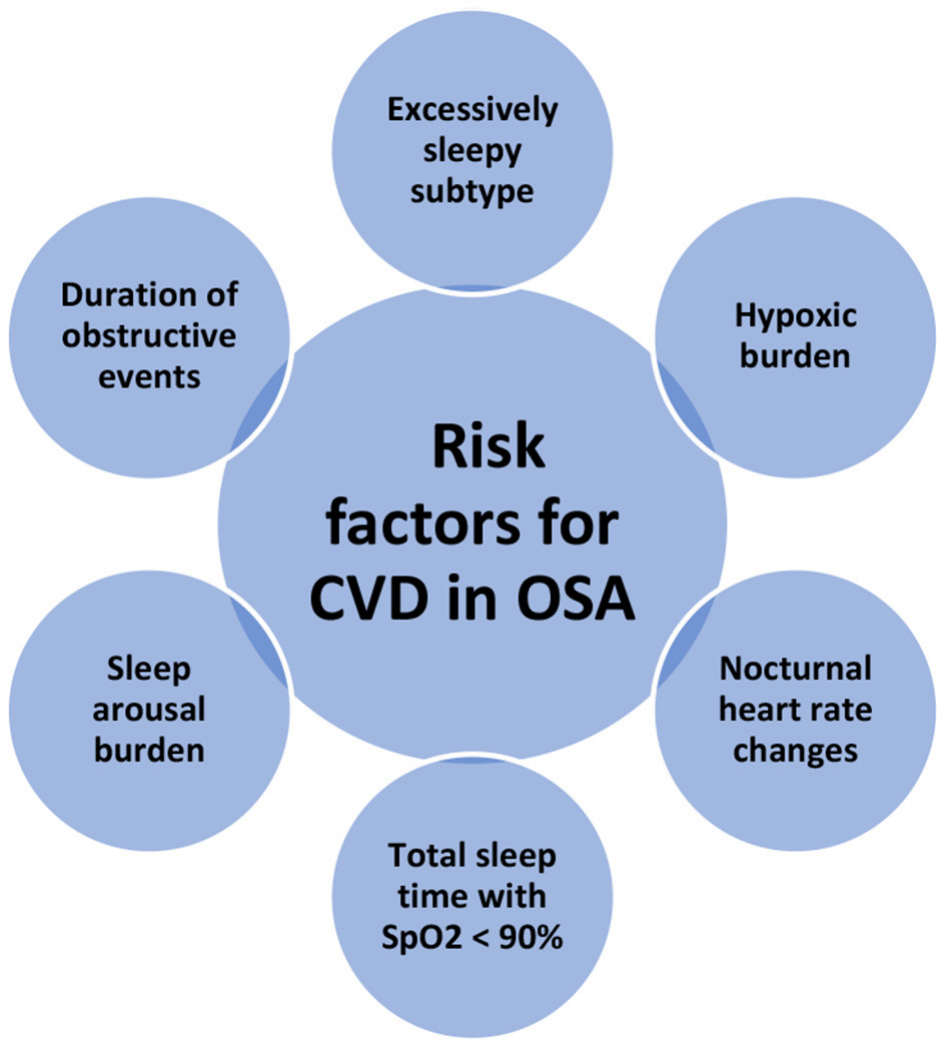
Variables impact the relationship between OSA and cardiovascular disease (CVD).

#### Hypoxic Burden

Another important variable that influences cardiovascular risk in OSA patients is the level of hypoxia during sleep. Recent data suggested that CVD mortality is strongly associated with a quantitative measure of the hypoxic burden. Specifically, Labarca et al. (31) showed a substantial difference between all-cause mortality in patients with moderate to severe OSA and hypoxic features. Likewise, Azarbarzin et al. (32) demonstrated that OSA severity quantified as the respiratory event-associated hypoxic burden was independently associated with CVD mortality. In contrast, CVD mortality was not associated with AHI when assessed as an independent predictor of mortality.

#### Nocturnal Heart Rate Changes

Two independent studies assessed different prospective community-based cohorts, found that the nocturnal heart rate changes (frequency of heart rate changes throughout sleep) predicted adverse cardiovascular morbidity and mortality (26, 33). The relatioship between heart rate changes during sleep and adverse cardiac outcome remained significant after adjusting for demographic factors, hypoxia, and other comorbidities (26). In another pilot study, Sankari et al. (34) non-apneic respiratory events without cortical arousal or desaturation were associated with significant heart rate changes, and optimal CPAP not only reduced the resistive load but normalized heart rate indicating that heart rate changes may play an essential role in the cardiovascular outcome in patients with OSA.

#### Total Sleep Time With SpO2 <90% (TST90)

Another hypoxia measure commonly obtained in sleep studies is O2 saturation total sleep time below 90%. Oldenburg et al. (35) showed the amount of TST90 better predicted all-cause mortality in OSA patients with heart failure even after adjustment for confounding factors. In their study, the risk of death increased by 16% for every hour of TST90.

#### Duration of Obstructive Events

The duration of respiratory events is readily available but rarely assessed for its clinical value or interpretation of sleep studies. Recently, Butler et al. (36) found that shorter obstructive events predicted all-cause moral-cause and beyond the AHI. Event duration is a heritable trait reflecting arousability.

#### Sleep Arousal Burden

Insufficient sleep is associated with an increased risk for cardiovascular disease and mortality. Assessing the clinical burden of inadequate sleep using a rate of arousals and the duration of individual arousal events called arousal burden may enhance our ability to risk-stratify patients with OSA and increase the risk for cardiovascular disease. Shahrbabaki et al. (37) found that nocturnal arousal burden is associated with long-term cardiovascular and all-cause mortality in women and to a lesser extent in men.

#### microRNA

Recent advances in the field of genomic research have helped in identifying non-coding RNAs. Expression of these non-coding RNAs was found to change in conditions such as cardiac hypertrophy, heart failure, and cardiac remodeling, reflecting their significance as diagnostic and prognostic biomarkers (38). The assessment of microRNAs and exosomes has also led to important insights both in terms of OSA biomarkers and potential therapeutic targets addressing OSA complications like CVD (39–43).

## The Future

Although the AHI is easy to use, this measure discounts other physiological consequences of the respiratory events that may be important, including associated hypoxemia and arousals from sleep and the cardiac autonomic disturbances throughout the night (48). Increasing interest in understating the precision medicine related to SDB and its cardiovascular consequences requires further investigation. These opportunities include the pathophysiology, diagnosis, and management of SDB in this population.

**Figure 2:**
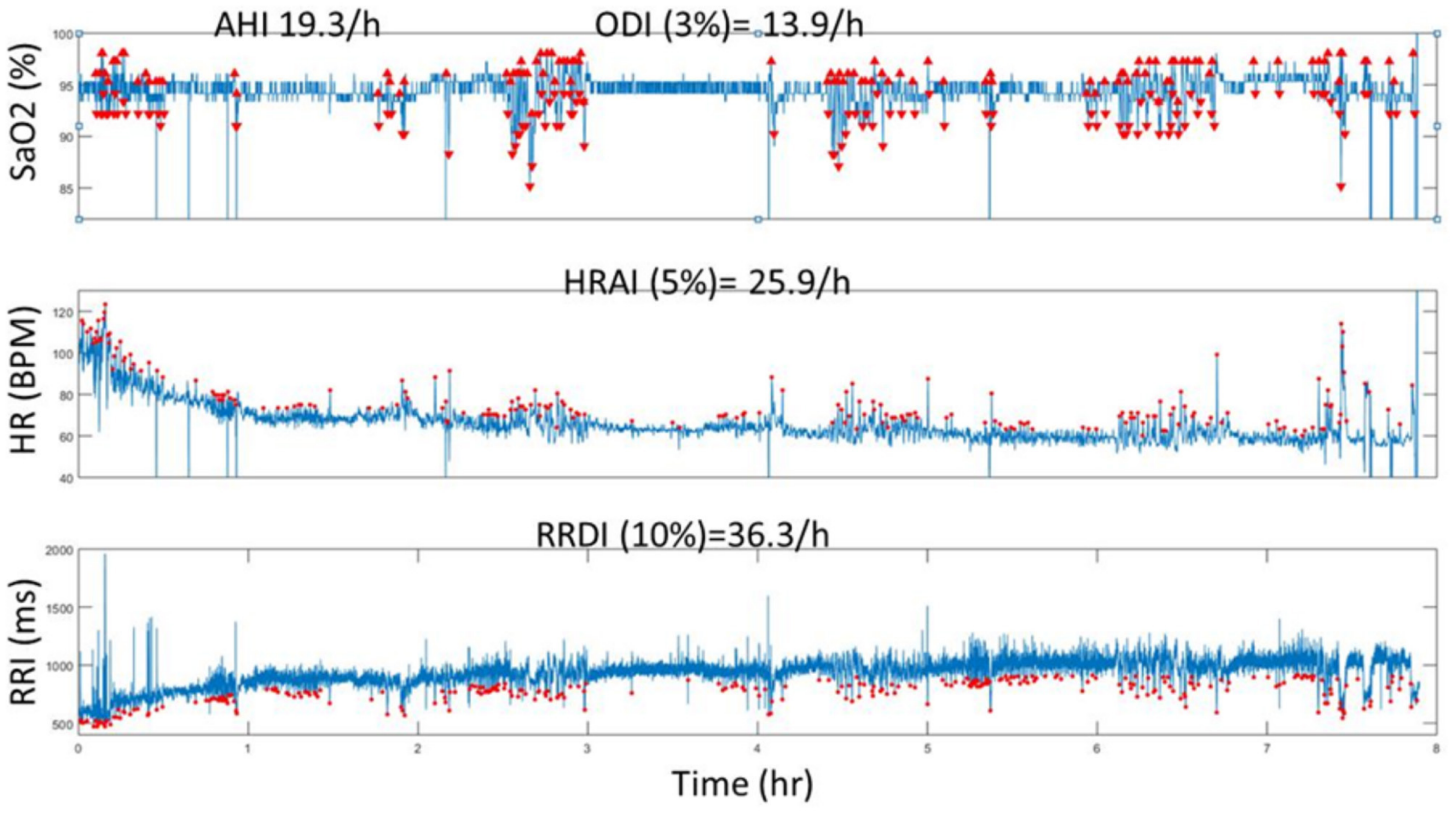
A representative computed data (Tachogram) using automated analysis of SaO2, HR and RRI from one individual who has SDB (AHI = 19.3 events/h). The red dots represent the O2 deasturations, pulse rate accelerations (HR) and RRI dips (from ECG) throughout the duration of the PSG recording (8 h). Note the incremental increase of values from ODI, AHI to HRAI and RRDI. HR, heart rate dervied from pulse signal; HRAI, pulse rate acceleration index; RRI, RR interval; RRDI, RRI dips index; SaO2, oxygen ssaturation.

• Physiological variables: Current hypopnea definitions are based on physiologic consequences of decreased flow, such as oxyhemoglobin desaturation or EEG arousals. The original definition of hypopnea included flow reduction associated with subsequent oxyhemoglobin desaturation ranging from 2 to 5% (44, 45). In a recent study, we and others found that heart rate indexes (change in heart rate and R-R interval dips index) were associated with increased rates of adverse cardiovascular outcomes in large prospective cohorts (26, 33). Therefore, incorporating physiological metrics such as heart rate in the diagnosis of SDB may enhance the precision of identifying a clinically significant disease. Figure 2 depicts the relationship between three physiological metrics in a patient with OSA.

• Clinical symptoms: It is suspected that one of the main reasons for the failure of recent clinical trials to demonstrate a significant effect on the reduction in cardiovascular risk with PAP therapy could be due to the exclusion of patients with excessive daytime sleepiness. Therefore, including sleepiness as a marker of clinically significant disease and not only measuring severity of disease based on the apnea-hypopnea index when assessing cardiovascular outcome in clinical trials of OSA treatment is warranted for further investigation in the future.

• Genetic and biomarkers testing. Although OSA has long been considered a heritable trait, few studies have looked at the genetic causes of OSA in recent years (39, 46). Very recently, there have been several investigations on the role of microRNAs (miRNAs) as potential markers for cardiovascular disease (47). Specifically, scientists have proposed to use miRNAs in the diagnosis and prognosis of cardiovascular disease (42). More importantly, measuring circulating serum levels of miRNAs helps identify individuals with OSA at risk of CVD and could become a therapeutic target for cardiovascular diseases. Further investigations of the genetic factors of OSA are urgently needed.

Nevertheless, there is a need for further investigation and development in this domain of cutting-edge technology.

- Wearable technologies.

While there has been a revolution in several wearable devices available for tracking activity and other physiological metrics during wake and sleep, several issues and challenges need to be overcome before clinical adaptation has become ideal. One major issue is access to internal raw data to evaluate its accuracy and validation and the issue of inter-device reliability and ability to interact with electronic medical records and sleep centers platforms.

- Machine learning and artificial intelligence technologies.

Artificial intelligence and machine learning algorithms have become a natural next step for personalized medicine with the explosion of digitalized healthcare information. While there is recent effort to use neural network learning to diagnose sleep apnea in children, the effort remains very limited to its potential impact and need. In futuristic aspiration, combining all the above opportunities (physiological/clinical variables, biomarkers, and data from wearables) into one machine learning platform could help identify and treat OSA cardiac consequences early and perhaps save lives and become a cost-saving method.

## Conclusions

Cardiac consequences of SDB are expected. However, the heterogeneity in identifying clinically significant responses to therapy is likely due to the lack of a complete clinical picture of these patients and the gap in our understanding of the disease. The current diagnostic approach of SDB needs to be challenged with a personalized approach and big data. The targeted and customized therapies aiming to identify high susceptibility to CVD are sorely required in patients with OSA and may impact their outcome. Nevertheless, none of these interventions have been tested systematically in patients with OSA.

## Author Contributions

AS contributed to the concept of the study and provided the future concept in the field. AP reviewed the past section of the research work. MY reviewed the current research work. AS, AP, and MY wrote sections of the manuscript. All authors contributed to the article and approved the submitted version.

## Conflict of Interest

The authors declare that the research was conducted in the absence of any commercial or financial relationships that could be construed as a potential conflict of interest.

## Publisher's Note

All claims expressed in this article are solely those of the authors and do not necessarily represent those of their affiliated organizations, or those of the publisher, the editors and the reviewers. Any product that may be evaluated in this article, or claim that may be made by its manufacturer, is not guaranteed or endorsed by the publisher.
